# Rare case of myelodysplastic syndrome with excess blasts 2 developing after adjuvant chemoradiotherapy for triple-negative breast cancer in a patient with Bloom syndrome

**DOI:** 10.1007/s00066-024-02257-z

**Published:** 2024-07-12

**Authors:** Ali Fuat Gürbüz, Melek Karakurt Eryılmaz, Oğuzhan Yıldız, Fahriye Kılınç, Murat Araz, Mehmet Artaç

**Affiliations:** 1 Department of Medical Oncology, Faculty of Medicine, Necmettin Erbakan University, 14280 Konya, Turkey; 2 Department of Pathology, Faculty of Medicine, Necmettin Erbakan University, Konya, Turkey

**Keywords:** Bloom syndrome, Triple negative breast cancer, Myelodysblastic syndrome with excess blasts 2, Adjuvant chemoradiotherapy

## Abstract

**Introduction:**

Bloom syndrome (BS) is a rare autosomal recessive disorder caused by a loss-of-function mutation in the *BLM* gene encoding an RecQ helicase involved in DNA repair and maintenance of chromosomal stability. In patients with BS, significant sensitivity to both DNA-damaging chemotherapy (CT) and ionizing radiation complicates the management of neoplasms by exacerbating comorbidities and predisposing to toxicities and poor outcomes.

**Case report:**

A 30-year-old female patient diagnosed with BS who presented with early-stage triple-negative breast cancer was treated with four cycles of doxorubicin (60 mg/m^2^) and cyclophosphamide (600 mg/m^2^) followed by weekly paclitaxel (80 mg/m^2^) for 12 weeks as the chemotherapy protocol and a total of 5000 cGy curative radiotherapy (RT). Due to pancytopenia 8 months after completion of therapy, bone marrow biopsy and aspiration were performed, and a diagnosis of myelodysplastic syndrome with excess blasts 2 (MDS-EB2) was made. Two courses of the azacitidine (75 mg/m^2^) protocol were administered every 28 days in the hematology clinic. Two weeks after CT the patient was transferred from the emergency department to the hematology clinic with the diagnosis of pancytopenia and febrile neutropenia. She died at the age of 33 due to sepsis that developed during follow-up.

**Conclusion:**

Due to the rarity of BS, there is no prospective trial in patients with cancer and no evidence base upon which to design treatment programs. For these reasons, it is strongly recommended that patients receive multidisciplinary care, with precise assessment and discussion of the indication and an adequate dose of DNA-damaging agents such as chemotherapy and ionizing radiation.

## Introduction

Bloom syndrome (BS) is a rare autosomal recessive disorder caused by a loss-of-function mutation in the *BLM* gene, which encodes an RecQ helicase involved in DNA repair and maintenance of chromosomal stability. Defective BLM protein results in replication errors with high rates of chromosomal rearrangements and breakage, leading to premature aging, early onset of age-related diseases, and a variety of early-life malignancies [1–4]. In patients with BS, significant sensitivity to both DNA-damaging chemotherapy (CT) and ionizing radiation complicates the management of these neoplasms by exacerbating comorbidities and predisposing to toxicities and poor outcomes [5]. The clinical picture often includes short stature, facial rash, and recurrent infections due to severe immunodeficiency. Genomic instability is also manifested by an increased susceptibility to a variety of cancers, particularly hematologic and gastrointestinal neoplasms [6, 7].

Herein, we report our experience in the management of breast cancer and myelodysplastic syndrome with excess blasts 2 (MDS-EB2) in a patient with BS.

## Case report

A 30-year-old premenopausal woman with BS was diagnosed with classic symptoms of short stature, growth retardation, and prominent facial features, as well as a homozygous deletion of exon 11,12 (mutation BLM gen region) detected in genetic analysis at the age of 14. The patient’s body mass index (BMI) was between 18 and 21 kg/m^2^, and she had minimal subcutaneous fat. At the age of 14, she developed combined variable immunodeficiency, which was treated with monthly intravenous immunoglobulin (IVIG). The patient presented to our outpatient clinic in April 2020 with a palpable mass in the left axilla that had been present for approximately 5 months. Superficial ultrasonography (USG) revealed a 5 × 3 cm mass in the left axillary tail and a 5 × 2 cm solid cystic nodule in the right thyroid lobe. The lesion in the left axillary tail was excised and sentinel lymph node sampling was performed. Histology reported an invasive ductal carcinoma, hormone receptor (HR) negativity, human epidermal growth factor receptor 2 (HER2) negativity, and a Ki-67 index of 60% and one lymph node removed is reactive. At the time of diagnosis, the level of the tumor marker CA15.3 was higher than normal (41 U/ml [0–27.9]), while the levels of CEA and CA125 were normal (1.3 ng/ml [0–5.2] and 31 U/ml [0–35]). Postoperatively, breast USG showed a benign hypoechoic lesion measuring 10 × 5 mm in the upper external quadrant of the right breast, and magnetic resonance imaging (MRI) showed a lesion in the right breast consistent with a fibroadenoma. No lesion was found to be the primary focus. The fluorodeoxyglucose positron-emission tomography/computed tomography (FDG-PET-CT) scan revealed no malignancy except for a hypodense nodule in the right lobe of the thyroid that did not retain FDG. The biopsy of the nodule in the right thyroid lobe yielded results confirming that it was benign. The pathological staging was T2N0. The patient was diagnosed with early-stage triple-negative breast cancer by the multidisciplinary pathologic oncology council.

**Fig. 1:**
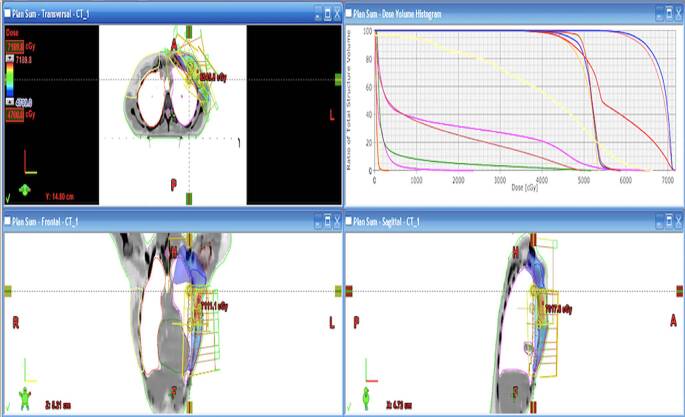
Radiotherapy (*RT*) dose planning and left chest wall and axilla using the radiotherapy scheme for the chest wall and axillatransversal section images on the top left, frontal section images on the bottom left, sagittal section images on the bottom right and dose-volume histogram on the top right

**Fig. 2:**
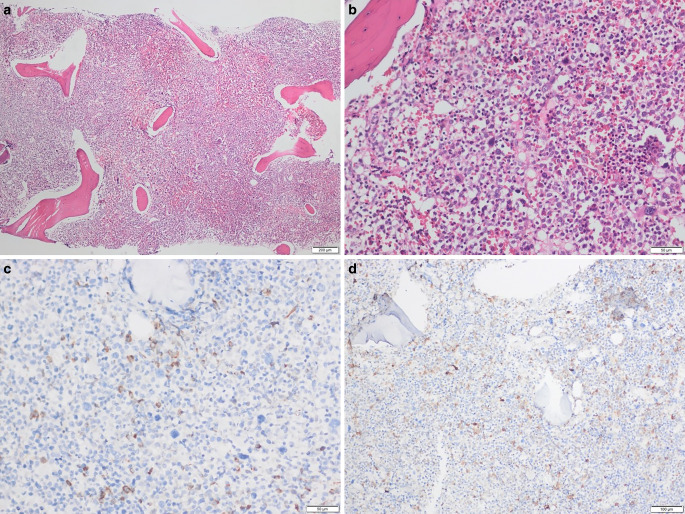
**a** Bone marrow biopsy showing a hypercellular appearance (hematoxylin/eosin; 40 × magnification). **b** Cell aggregates with large nuclei, some with prominent nucleoli and hypolobulated megakaryocytes (hematoxylin/eosin; 200 × magnification). **c** Immunohistochemical CD34 staining shows an increased number of blastic cells reacting (CD34; 200 × magnification). **d** Increased immunohistochemical CD117 positivity is observed in blastic cells (CD117; 100 × magnification)

The patient was scheduled to receive adjuvant treatment consisting of four cycles of doxorubicin (60 mg/m^2^) and cyclophosphamide (600 mg/m^2^) followed by weekly paclitaxel (80 mg/m^2^) for 12 weeks. The echocardiogram demonstrated a left ventricular ejection fraction (LVEF) of 60%, and no evidence of valve pathology was observed. It was recommended that the patient continue the current intravenous immunoglobulin (IVIG) treatment. Following completion of the computed tomography (CT) plan, no malignant lesions were identified in the control positron-emission tomography (PET) and breast ultrasound (US) examinations. The patient received 25 fractions of 200 cGy (2 Gy) daily to the left chest wall and axilla using the intensity-modulated radiation therapy (IMRT) technique. A total of 5000 cGy (50 Gy) curative radiotherapy (RT) was planned with fractionation, and a boost was performed in 8 fractions (Fig. 1). Due to pancytopenia 8 months after completion of therapy, bone marrow biopsy and aspiration were performed, and a diagnosis of myelodysplastic syndrome with excess blasts 2 (MDS-EB2) was made (Fig. 2). Two courses of the azacitidine (75 mg/m^2^) protocol were administered every 28 days in the hematology clinic. Two weeks after the CT scan, the patient was transferred from the emergency department to the hematology clinic with a diagnosis of pancytopenia and febrile neutropenia. She died at the age of 33 due to sepsis that developed during follow-up.

## Discussion

Bloom syndrome is defined by a number of characteristics, including growth retardation, immunodeficiency, early development of age-related comorbidities, a predisposition to early and varied cancers, and increased sensitivity to drug toxicity [8]. In general, leukemia and lymphoma are the most prevalent neoplasms in individuals with BS. Among solid tumors, colorectal and other gastrointestinal cancers are common, but cancer of almost all organ systems has been observed [9]. In a prospective study by Sugranes TA et al., among the 290 individuals with BS, 155 developed cancer, with 100 (65%) diagnosed with a single cancer. The remaining 55 patients (35%) developed multiple cancers, with an average interval of 6 years between the first and second malignancies. Among 251 neoplasms, 83 (33%) were hematologic and 168 (67%) were solid tumors. Hematologic malignancies were more prevalent than any of the solid tumors. The most frequently observed solid tumors were colorectal, breast, and oropharyngeal. The second most common malignancies were leukemia and breast cancer, each present in 10 patients (18%). The observed cases of acute myeloid leukemia occurred subsequently to chemotherapy treatments for the primary cancer and were typically preceded by myelodysplastic syndrome. The cumulative incidence of any malignancy by age 40 was 83%. The median survival for all participants was 36.2 years [10]. Similarly, the patient with BS developed both breast cancer and MDS-EB2 during the follow-up period, with an overall survival of 33 years. In contrast, the time to the development of a secondary malignancy was remarkably brief, with a median of only 8 months.

In the literature review, case reports of malignancies accompanying BS are usually related to hematological malignancies or solid cancers [11, 12]. A single case report exists in which hematological and solid cancers were observed in the same individual [13]. This case report represents the second such instance.

The oncogenic potential of CT and radiation exposure constitute a challenge for cancer treatment among patients with BS. However, due to the rarity of BS, the existing literature is limited. The paucity of data concerning cancer treatment in patients with BS, coupled with the absence of clear guidelines for their management and treatment adjustment, represents a significant challenge for physicians tasked with their care. A recent published article by several patient societies emphasized the importance of a BS-adapted approach for the BS community [14]. Despite the lack of systematic studies on the treatment of malignancies in BS, most treating physicians have employed reduced or omitted (weight-based) doses of CT and RT to avoid excessive toxicity.

Although there are no evidence-based data linking specific chemotherapeutic agents to an increased risk of secondary malignancies, most treating clinicians recommend against the use of alkylating agents, particularly busulfan, cyclophosphamide, or melphalan, due to their direct interaction with DNA. Only in vitro studies have demonstrated that the introduction of 5‑fluorouracil into DNA results in greater DNA fragmentation in cells with pathogenic variants of BLM compared to control cells. A comparable concern pertains to radiation exposure in the diagnosis and treatment of patients with BS. Irradiated Blm-deficient mice (Blmm3/m3) exhibited an increased risk for tumors, particularly hematological malignancies [15, 16].

Individuals with DNA repair disorders are at an increased risk of developing malignant tumors and of hypersensitivity to radiotherapy. These tumors commonly appear at an early age and have a poor prognosis [17]. Similarly, there is a lack of evidence-based data on the use of chemotherapeutic agents in conjunction with RT. In the international medical literature, radiotherapy (RT) in patients with BS and solid cancers is only rarely described in case reports [18–21]. Between the 1950s and 1970s, a total of 14 patients with acute leukemia were identified in the German registry. Seven of these patients developed severe treatment reactions, including fatal bone marrow suppression, interstitial pneumonitis and hepatitis, mucositis leading to severe intestinal hemorrhage, candidiasis, and neurological toxicity. Some of these reactions occurred despite reduced doses of chemotherapy. The remaining patients did not exhibit any unusual reactions, although the data for some patients are limited, and only two survived their disease and treatment [22]. In contrast to these studies, our patient did not develop any serious complications during RT. The data indicate that there is no definitive evidence to suggest that CT or RT can be linked to an increased risk of malignancy. However, case reports suggest that these treatments have the potential to induce malignant transformation, a possibility that should be kept in mind when assessing individual cases.

Our patient had an aggressive triple-negative histology and was diagnosed at an early age. We therefore predicted that she would have a poor prognosis, as in a study by Kanyılmaz et al. involving 559 patients, the 5‑year overall survival rate of patients with triple-negative breast cancer was significantly lower than that of patients with hormone receptor-positive and HER2-positive breast cancer. In addition, patients with triple-negative disease had a 2.64 times increased risk of death compared to other groups (hazard ratio: 2.64, 95% confidence interval [1.36–5.12]; *p* = 0.004), [23]. We had to use alkylating agents and RT, which are recommended in current guidelines for the triple-negative subtype. After a multidisciplinary tumor board discussion, we planned adjuvant CT followed by RT with no dose reduction. Although dose reduction is recommended in the literature based on case reports rather than evidence-based randomized treatments, we treated at standard doses and achieved an oncological response.

## Conclusion

Current studies have observed the development of multiple cancers with an average of many years between the first and second malignancies. Our case is rare due to the short interval between the development of secondary malignancy and aggressive progression. Due to the rarity of BS, there are no prospective studies in patients who develop cancer and there is no evidence base to guide treatment programs. As there is no consensus on optimal treatments, physicians encounter significant difficulties in managing patients with this condition. For these reasons, it is strongly recommended that patients receive multidisciplinary care, with careful evaluation and discussion of the indication and an appropriate dose of DNA-damaging agents, such as chemotherapy and ionizing radiation therapy.
